# AraDQ: an automated digital phenotyping software for quantifying disease symptoms of flood-inoculated *Arabidopsis* seedlings

**DOI:** 10.1186/s13007-024-01171-w

**Published:** 2024-03-16

**Authors:** Jae Hoon Lee, Unseok Lee, Ji Hye Yoo, Taek Sung Lee, Je Hyeong Jung, Hyoung Seok Kim

**Affiliations:** 1 Department of Agricultural Biotechnology, Seoul National University, Seoul, 08826 Republic of Korea; 2 Research Institute of Agriculture and Life Sciences, Seoul National University, Seoul, 08826 Republic of Korea; 3 Smart Farm Research Center, Korea Institute of Science and Technology, Gangneung, 25451 Republic of Korea

**Keywords:** *Arabidopsis*, Deep learning, Digital phenotyping, Disease quantification, Image analysis, *Pseudomonas syringae*

## Abstract

**Background:**

Plant scientists have largely relied on pathogen growth assays and/or transcript analysis of stress-responsive genes for quantification of disease severity and susceptibility. These methods are destructive to plants, labor-intensive, and time-consuming, thereby limiting their application in real-time, large-scale studies. Image-based plant phenotyping is an alternative approach that enables automated measurement of various symptoms. However, most of the currently available plant image analysis tools require specific hardware platform and vendor specific software packages, and thus, are not suited for researchers who are not primarily focused on plant phenotyping. In this study, we aimed to develop a digital phenotyping tool to enhance the speed, accuracy, and reliability of disease quantification in *Arabidopsis*.

**Results:**

Here, we present the Arabidopsis Disease Quantification (AraDQ) image analysis tool for examination of flood-inoculated *Arabidopsis* seedlings grown on plates containing plant growth media. It is a cross-platform application program with a user-friendly graphical interface that contains highly accurate deep neural networks for object detection and segmentation. The only prerequisite is that the input image should contain a fixed-sized 24-color balance card placed next to the objects of interest on a white background to ensure reliable and reproducible results, regardless of the image acquisition method. The image processing pipeline automatically calculates 10 different colors and morphological parameters for individual seedlings in the given image, and disease-associated phenotypic changes can be easily assessed by comparing plant images captured before and after infection. We conducted two case studies involving bacterial and plant mutants with reduced virulence and disease resistance capabilities, respectively, and thereby demonstrated that AraDQ can capture subtle changes in plant color and morphology with a high level of sensitivity.

**Conclusions:**

AraDQ offers a simple, fast, and accurate approach for image-based quantification of plant disease symptoms using various parameters. Its fully automated pipeline neither requires prior image processing nor costly hardware setups, allowing easy implementation of the software by researchers interested in digital phenotyping of diseased plants.

**Supplementary Information:**

The online version contains supplementary material available at 10.1186/s13007-024-01171-w.

## Background

The *Arabidopsis thaliana*–*Pseudomonas syringae* pathosystem has been widely used as a model for studying plant immunity and plant–microbe interactions. Extensive studies on their tug-of-war have provided a basis for unraveling the complexity of plant defense mechanisms. *P. syringae* is a widespread and agriculturally important plant pathogen that comprises more than 60 pathovars, a few of which, including *P. syringae* pv. *tomato* (*Pst*) DC3000 and *P. syringae* pv. *maculicola*, are pathogenic to *Arabidopsis* [1, 2]. The pathogen enters plants through stomatal openings and colonizes the apoplastic space. The characteristic disease symptoms in *Arabidopsis* include leaf yellowing, necrosis, and transient water-soaked spots during the early stages of infection [3, 4]. In general, plants mount multiple layers of innate immune responses to defend themselves against pathogen attacks, leading to stomatal closure, reactive oxygen species (ROS) burst, callose deposition, and sometimes, rapid cell death at the site of infection [5]. These factors collectively create a hostile environment for microbial growth. Consequently, plant pathogens have evolved several mechanisms for utilization of various virulence factors that counteract plant defenses. During infection, *Pst* DC3000 produces the phytotoxin coronatine that inhibits stomatal closure [6, 7]. Many bacterial species, including *Pst* DC3000, also deliver type III effectors into the plant cytoplasm to subvert host cellular processes for their own benefit [8].

To enhance our understanding of plant–microbe interactions, it is highly desirable to develop methods for the precise evaluation of disease severity in a high-throughput manner. Conventional inoculation methods, including syringe/vacuum infiltration, foliar spray, and dipping, are hardly applicable for high-throughput assays because of the difficulties in handling many samples at a time. On the other hand, the flood-inoculation method, which was first introduced as an assay for assessment of virulence of *Pst* DC3000 in tomato seedlings, was subsequently applied to *Arabidopsis* and has several advantages over the other methods in terms of simplicity, efficiency, and reliability [9, 10]. Moreover, this method allows uniform treatment and minimal mechanical wounding during inoculation, as well as relatively strict regulation of environmental conditions during the study period, since the only prerequisite for this method is that seedlings grown on solid medium in Petri plates should be submerged in a bacterial solution. For inoculation using conventional methods, 4- to 5-week-old plants are generally used, whereas 2-week-old seedlings are used for the flood-inoculation method, allowing the total set of experiments (from sowing to performing virulence assays) to be completed within 3 weeks.

Previous studies have shown that the flood-inoculation method can be effectively used to compare the effects of different plant lines and bacterial strains on disease development [11–14]. However, like that for other inoculation methods, a limitation of this method is that no proper quantification technique has been devised to measure disease severity at the phenotypic level, and thus, further verification steps need to be performed. Although bacterial growth assays have been widely used to evaluate plant disease severity, labor-intensive procedures such as homogenization and dilution plate counting often cause problems with reproducibility of the results [15, 16]. To overcome these limitations, several attempts have been made to develop reliable and simple quantification methods. For example, to examine bacterial growth *in planta*, a bioluminescence assay was conducted using chromosomally tagged *P. syringae* strains that constitutively expressed the *lux* operon [17]. Furthermore, quantitative real-time polymerase chain reaction (qRT-PCR) analysis of *P. syringae* genes was performed using DNA templates extracted from diseased plants [16]. However, for analysis using these approaches, the destruction of plant samples prior to performing the assays and/or the use of certain optical instruments and reagents is required.

Image-based phenotyping is an attractive alternative approach for quantifying plant disease symptoms. Non-invasive evaluation of plants via automated image-processing tools can provide an opportunity to expand the scale and the depth of analysis [18, 19]. Two-dimensional top-view images captured by a digital red, green, and blue (RGB) color camera are sufficient for phenotyping features such as growth and greenness of *Arabidopsis* rosettes. Furthermore, other sensor devices, including three-dimensional scanning systems and cameras for chlorophyll-fluorescence, thermal, and hyperspectral imaging, could be implemented to detect changes in not only anatomical but also physiological properties of plants during disease development [20–23]. Diverse image analysis tools have been developed to extract biologically relevant information from *Arabidopsis* rosette images, and those developed specifically for disease quantification purposes include PhenoPhyte (web-based tool for measurement of green plant area) and PIDIQ (ImageJ-based tool for measurement of chlorotic plant area) [15, 22, 24–26]. Despite the impressive quality of their performance, most of these tools have some limitations such as the requirement of specific hardware platforms, programming/scripting languages, or vendor lock-in tools with undocumented black box procedures. These image processing pipelines also lack provisions for adjustment of variations in scale and illumination, resulting in low batch-to-batch data consistency [27]. Most importantly, plant image segmentation of non-green components, for which accuracy is critical in ensuring reliable measurement of various parameters, remains challenging, especially if leaves turn yellow-white and dry up after inoculation.

In recent years, a wide range of long-standing problems in the field of computer vision have been revolutionized by deep learning technology [28–31]. The accuracy of pixel-wise classification tasks, including object segmentation, has been remarkably improved using deep convolutional encoder–decoder networks [32–34]. These networks facilitate end-to-end training because the encoder extracts low-resolution feature maps, and the decoder reconstructs them to the original input resolution. Some of the networks are particularly adapted for delineating the boundaries of small objects with the help of variations in color, and thus, these are promising resources for medical and aerial image analyses [35–37]. Problems in the segmentation of images of diseased *Arabidopsis* seedlings have been caused by similarities in semantic features of thin petioles and discolored leaves, and these problems can be effectively addressed using the deep neural approach.

In this study, we aimed to develop a digital phenotyping tool to enhance the speed, accuracy, and reliability of *Arabidopsis* disease quantification. We trained deep learning models to achieve a high degree of performance in terms of object detection and segmentation. Here, we present the Arabidopsis Disease Quantification (AraDQ) software, which enables the calculation of four different color parameters (green chromatic coordinate [GCC], excess green index [ExGI], green and red ratio vegetation index [GRVI], and hue saturation value [HSV]-based green/yellow color categorization) and six different morphological parameters (perimeter, projected leaf area, convex hull perimeter, convex hull area, compactness, and stockiness) for individual seedlings in the given image. We conducted two case studies using bacterial and plant mutants and demonstrated that this software is easily applicable for comparison of disease symptoms using solely the images of seedlings taken before and after treatment. The simple requirements for image preparation, straightforward interface, and versatile analytical capabilities are of great benefit to a wide range of studies on plants. Using the information obtained from the results of the flood-inoculation assay, AraDQ has the potential to enhance our understanding of various plant–microbe and plant-environment interactions.

## Materials and methods

### Plant materials and growth conditions

The genetic background of all mutant lines used in this study was the *A. thaliana* ecotype Columbia (Col-0). The *fls2*, *efr1*, and *fls2*/*efr1*/*cerk1 Arabidopsis* lines were gifted by Dr. Man-Ho Oh (Chungnam National University). For the flood-inoculation assays, the seeds were surface-sterilized using 70% ethanol for 1 min and 12.5% sodium hypochlorite containing 0.1% Tween 20 for 15 min and then washed with sterile distilled water. The sterilized seeds were plated on half-strength Murashige and Skoog (MS) basal medium supplemented with 0.05% 2-(*N*-morpholino)ethanesulfonic acid (MES), 1% sucrose, and 0.75% phytoagar in deep Petri dishes (100 mm diameter and 25 mm height), followed by 2 days of stratification at 4 °C. The plants were grown in a controlled growth room under a light intensity of 200 μmol m^−2^ s^−1^ and a 12 h light/12 h dark photoperiod at 22 °C for two weeks.

### Bacterial strains and growth conditions

*Pst* DC3000 strains harboring mutations in *hrpA* and *cmaA* were generated using the splice overlap extension mutagenesis as described previously [38, 39]. Briefly, 1-kb-long upstream and downstream regions of the gene to be deleted were amplified and assembled using the kanamycin resistance cassette of pKD13 by the overlap extension PCR method. The final PCR product was inserted into the SmaI-digested pTok2 vector and introduced into *Pst* DC3000 cells by electroporation. Transformants that were kanamycin resistant, but not tetracycline resistant, were selected and gene deletion was verified by PCR. The primer sequences used in this study are listed in Additional file 1. The bacterial strains were incubated at 28 °C on King’s medium B (KB) to which the following concentrations of antibiotics were added when required: 100 μg/ml rifampicin, 50 μg/ml kanamycin, and 15 μg/ml tetracycline.

### Image data acquisition

**Fig. 1:**
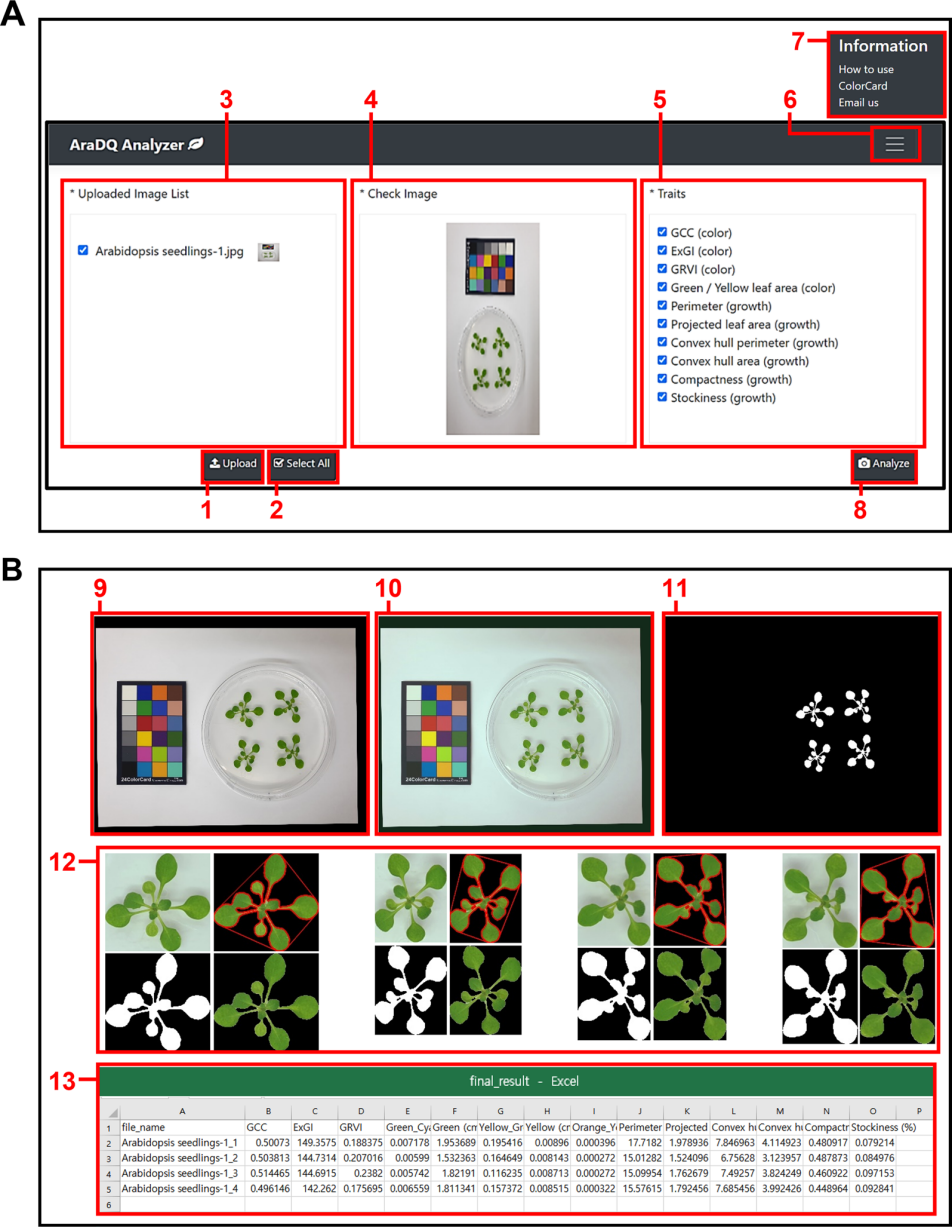
Graphical user interface and image processing using AraDQ. **A** Elements of the AraDQ graphical user interface. (1) Button for uploading images, (2) button for selection of all images uploaded, (3) window displaying list of images uploaded, (4) window displaying selected image, (5) window displaying traits to be quantified, (6) button for showing information menu, (7) additional window displaying links to user manual, color card information, and contact information, and (8) button for starting image analysis. **B** Results provided following AraDQ analysis. (9) distortion-corrected image, (10) color-corrected image, (11) binary image, (12) images of individual seedlings after distortion correction, color correction, binary segmentation, and trait quantification, (13) Trait quantification results are shown in a comma-separated values (CSV) format

Flood-inoculation assays were performed as previously described with a few modifications [10]. Briefly, bacterial cells were allowed to grow overnight and then suspended in distilled sterile water supplemented with 0.025% Silwet L-77 (PhytoTechnology Laboratories, Shawnee Mission, KS, USA); the suspension was adjusted to the appropriate concentration. The bacterial suspension was dispensed into a Petri plate containing 2-week-old *Arabidopsis* seedlings, and after 3 min, the suspension was removed by decantation. After inoculation, the Petri plates were sealed with 3 M Micropore 2.5-cm-wide surgical tapes (3 M, Jeonju, Korea) and incubated in a controlled growth room under a light intensity of 200 μmol m^−2^ s^−1^ and a 12 h light/12 h dark photoperiod at 22 °C for 3 days. A maximum of four *Arabidopsis* seedlings were grown in one Petri plate, as our system was designed to detect up to four individuals to avoid overlapping of leaves from neighboring seedlings. Images were taken with a 24-color balance card, 2 × 3 inches (CameraTrax, Las Vegas, NV, USA), placed on a white background for the use of the image processing model in AraDQ (Fig. 1).

**Fig. 2:**
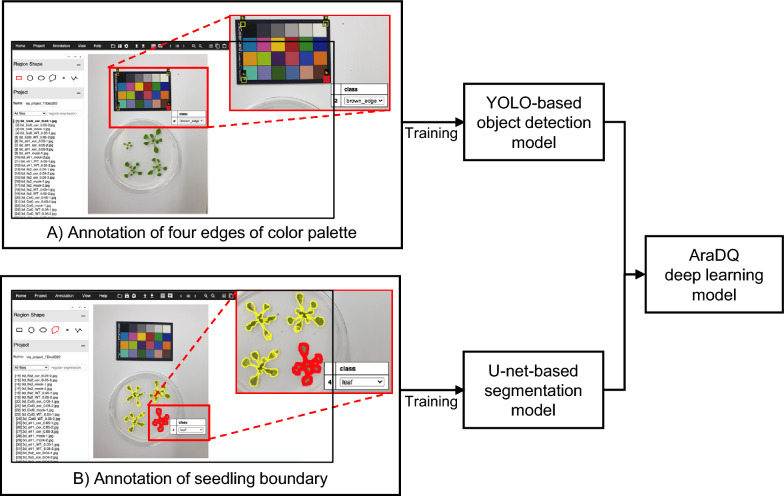
Deep learning models used in AraDQ.** A** Image annotation to train the You-Only-Look-Once (YOLO) model for detection of the four edges of the color palette. **B** Image annotation to train the U-net model for seedling segmentation

For the preparation of deep learning training images, the ground truth data were generated by manual labeling using the Visual Geometry Group (VGG) Image Annotator (VIA), which is an open-source image annotation software developed by VGG at the University of Oxford (Fig. 2) [40]. A total of 1,554 and 1,996 annotated images of the color palette and the seedlings, respectively, were obtained and randomly split into training (80%) and validation (20%) datasets.

## Implementation

### System architecture

AraDQ is a cross-platform application program with pretrained deep neural networks and a trait-quantification pipeline for *Arabidopsis* seedlings. It offers a graphical user interface (GUI) that allows ease of use and access to any user. The pipeline has been designed to automate and facilitate all image-processing steps once the inputs are set. AraDQ consists of three sections: (1) uploaded image list, (2) check image, and (3) traits (Fig. 1a). Digital images for the analysis can be provided through manual selection by clicking the “Upload” button. The names and contents of the successfully uploaded images are displayed in the left and the middle sections. The images to be processed and the traits of interest can be selected before analysis using checkboxes, thereby speeding up the data acquisition process as required. The outputs are stored in the form of comma-separated values (CSV) files and different types of processed image files, including distortion- and color-corrected images and binary images of the seedlings (Fig. 1b). The image files of individual seedlings generated following image processing and trait quantification are also provided for visual comparison (Fig. 1b). These files can be found in the folder named according to the date and the time of analysis (YYYY_MM_DD_HH_MM) under the “Results” folder in the same location where AraDQ is installed, so that the results can be organized in chronological order. The portable software, system code, and installation manual are available at https://github.com/kist-smartfarm/AraDQ.

### Image pre-processing and processing

Our AraDQ deep learning model involves two separate artificial intelligence models for object detection and segmentation, as shown in Fig. 2. For AraDQ, the input image should contain the indicated color palette with a known size and color distribution for standardized measurement of traits using different sources of datasets. The model trained using the You-Only-Look-Once (YOLO) version 4 (YOLOv4) network detects four edges of white, brown, black, and cyan patches in the palette, allowing distortion- and color-correction using projective transformation and gamma correction algorithms, respectively (Fig. 2a) [41–44]. Seedling segmentation is performed using the trained model with the customized U-net, where additional layers of batch normalization combined with early stopping are applied to avoid overfitting (Fig. 2b) [45].

**Fig. 3:**
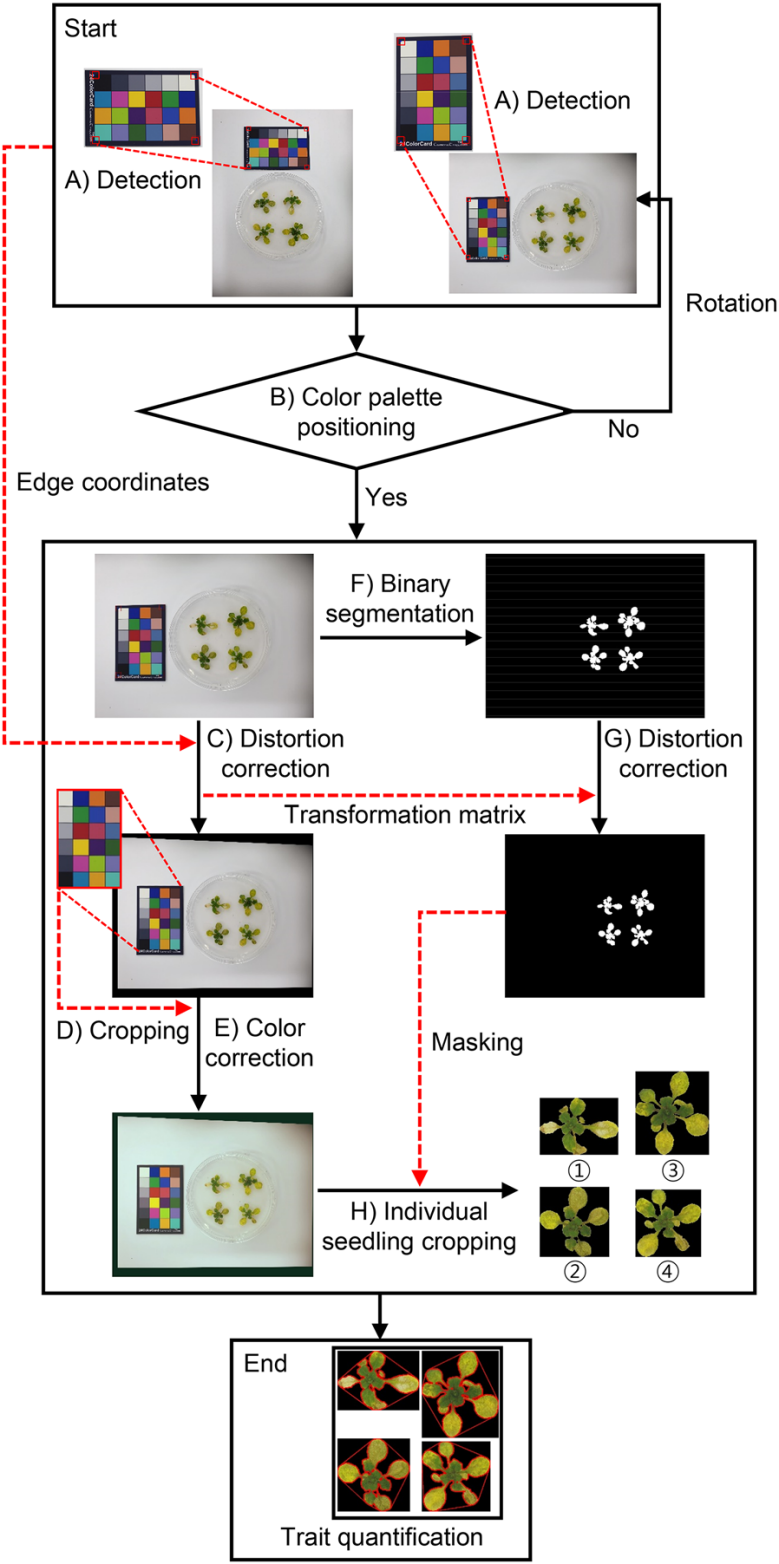
An overview of the image processing workflow in AraDQ.** A** Detection of four edges of the color palette. **B** Image rotation to place the white patch of the color palette near the top-left corner. **C** Distortion correction of original input image. **D** Cropping of color palette. **E** Color correction of distortion-corrected image. **F** Binary segmentation of seedlings. **G** Distortion correction of binary image. **H** Cropping images of individual seedlings for the subsequent trait quantification step

The overall workflow of the image processing in AraDQ is shown using an input image as an example in Fig. 3. The model first detected the color palette edge in the image (Fig. 3a) and rotated it to place a white patch near the top-left corner (Fig. 3b). Since the size of the color palette itself is not variable, the input image was then corrected based on projective transformation using the absolute coordinates of the color palette edges as a reference (Fig. 3c). The transformed color palette was cropped (Fig. 3d) and used for color correction using the gamma correction algorithm (Fig. 3e). Meanwhile, the input image was also converted to a binary format with the seedling and the background displayed in white and black, respectively, to isolate the regions of interest through the deep learning-based binary segmentation (Fig. 3f); the image was warped by the same transformation matrix that was used for distortion correction (Fig. 3g). The binary image was then used to extract the seedling parts from the color-corrected input image. Individual seedlings were isolated by a connected component labeling algorithm and numbered by increasing the Euclidean distances from the top-left corner of the image to the center-points of the seedlings, and subsequently, cropped in the form of a square image of the minimum size possible (Fig. 3h).

### Trait quantification

For digital phenotyping, AraDQ offers 10 different parameters (six and four associated with rosette morphology and color, respectively) that are useful for the quantification of plant disease severity [25, 26]. The morphological parameters included perimeter (cm), projected leaf area (cm^2^), convex hull perimeter (cm), convex hull area (cm^2^), compactness (%), and stockiness (%). Using the binary image border extraction algorithm, contour lines were extracted using the segmentation masks of each seedling [46]. The perimeter was determined by measuring the arc length for pixels along the contour, whereas the projected leaf area was determined by counting the number of pixels in the binary image. The convex hull for each seedling was computed using the Quickhull algorithm [47], and the perimeter and area were determined using the method described above. These values were converted to cm units using the pixel-to-cm conversion ratio; these calculations were performed using the fixed size of the color palette as a reference. Rosette compactness was calculated using the ratio between the projected leaf area and the convex hull area, and rosette stockiness was calculated using the following formula: 4 × π × projected leaf area/perimeter^2^ [48].

The color parameters included GCC, ExGI, GRVI, and HSV-based green/yellow color categorization (in cm^2^). The first three parameters are related to the greenness of leaves and were computed using the RGB color space as follows [49–52]:

$$GCC\, = \,\frac{{DN_{green} }}{{DN_{red} \, + \,DN_{green} \, + \,DN_{blue} }}$$

$$ExGI\, = \,2\,*\,DN_{green} \, - \,\left( {DN_{red} \, + \,DN_{blue} } \right)$$

$$GRVI\, = \,\frac{{DN_{green} \, - \,DN_{red} }}{{DN_{green} \, + \,DN_{red} }}$$

where DN denotes the digital number of red, green, and blue channels. The mean values of the total pixels for each seedling were also calculated. In addition, the segmentation images of each seedling were converted to the HSV color space, and the pixel numbers within different ranges of the hue channel (green-cyan class, 140° to 170°; green class, 81° to 140°; yellow-green class, 61° to 80°; yellow class, 51° to 60°; orange-yellow class, 41° to 50°) were counted for rating of the yellowing symptom [53]. This result was converted to cm units using the pixel-to-cm conversion ratio, as described above.

### Deep learning training and model evaluation

The training environment in this project was equipped with an Nvidia GeForce RTX 2070 8 GB GPU with a 32 GB RAM and an Intel Core i9-9900 K 3.60 GHz CPU. For color palette detection, the YOLOv4 model was trained in the Darknet framework with minor adaptations to resize the input of size 480 × 480 and detect the four classes (edges of white, brown, black, and cyan color patches) of the object [43]. Some hyperparameters such as subdivision [35], max batches (16,000), learning rate (0.00087), and steps (12,800 and 14,400) were modified based on the GPU performance. The model performance was evaluated by measuring the F_1_ score of the validation data for each epoch. The Jaccard index, also termed the Jaccard similarity coefficient and the Intersection over Union (IoU), was calculated as the intersection of the ground-truth bounding box with the predicted bounding box over the union. A true-positive (TP) was considered if the measured Jaccard index was ≥ 0.5; otherwise, it was considered as a false-positive (FP). Missed detection was considered as a false-negative (FN). The F_1_ score was calculated using precision (P) and recall (R), which indicated classifier exactness and completeness, respectively, as follows:

$$Precision\,\left( p \right)\, = \,\frac{TP}{{TP\, + \,FP}}$$

$$Recall \left( R \right) = \frac{TP}{{TP + FN}}$$

$${F}_{1} score=2 \cdot \frac{P\cdot R}{P+R}$$

Our trained object detection model exhibited stable and high level of performance (F_1_ score: 0.9725, precision: 0.9525, and recall: 0.995) after 3000 epochs.

The modified U-net model, consisting of four levels of depth with additional batch normalization on both the encoder and the decoder, was trained for seedling segmentation [45]. The model performance was evaluated by measuring the F_1_ score of the validation data for each epoch. In semantic segmentation, TP was defined as pixels correctly classified as objects, FP as pixels incorrectly classified as objects, and FN as pixels classified as objects in the ground truth but not classified as objects in the model prediction. The F_1_ score was calculated using precision and recall as described above. Our trained segmentation model exhibited stable and high level of performance (F_1_ score: 0.9915, precision: 0.9913, and recall: 0.9917) after 100 epochs.

### Case studies for validation of AraDQ performance

Two case studies were conducted to validate the performance of AraDQ, encompassing functions related to deep learning-based leaf segmentation and algorithm-based calculation of color parameters. Plant images were sourced from an independent experimental dataset that did not overlap with the images used to develop the deep learning model.

In Case Study I, a total of 114 *Arabidopsis* seedlings were utilized for flood-inoculation assays, incorporating three different bacterial strains and six distinct inoculum concentrations, including a control group. Each factor combination in this study had six replicates for each individual seedling.

In Case Study II, a total of 72 seedlings, consisting of four different plant mutants and wild-type plants, were subjected to assays involving three different bacterial treatments. Similar to Case Study I, there were six replicates for each factor combination.

It's important to note that the independent experimental dataset, non-overlapping with the images used for model development, was employed in both case studies to ensure the robustness and generalizability of AraDQ's performance evaluation.

## Results and discussion

### Case study: analysis of disease symptoms caused by different mutant strains

Characterization of the role of virulence factors in disease development is critical for understanding how pathogens establish parasitic relationships with their hosts. Pathogens have evolved different proliferation and survival strategies depending on their host range and lifestyle [54]. Comparative genomic analyses of several *P. syringae* strains have revealed that *Pst* DC3000 encodes a range of virulence factors, including effector proteins, phytotoxic compounds, cell wall-degrading enzymes, phytohormones, flagella, attachment factors, and siderophores [55, 56]. Each virulence factor has a different functional significance in pathogenesis, and their coordinated action at the appropriate time is required for full virulence [57]. There have been a number of studies examining plant innate immune responses against *Pst* DC3000 mutants defective in virulence factor(s) [11, 58–61]; however, the effects on plant growth and chlorosis have rarely been reported due to the lack of proper phenotyping methods. AraDQ enables the calculation of changes in rosette growth and greenness after pathogen attack, allowing the comparison of disease symptoms caused by different mutant strains.

**Fig. 4:**
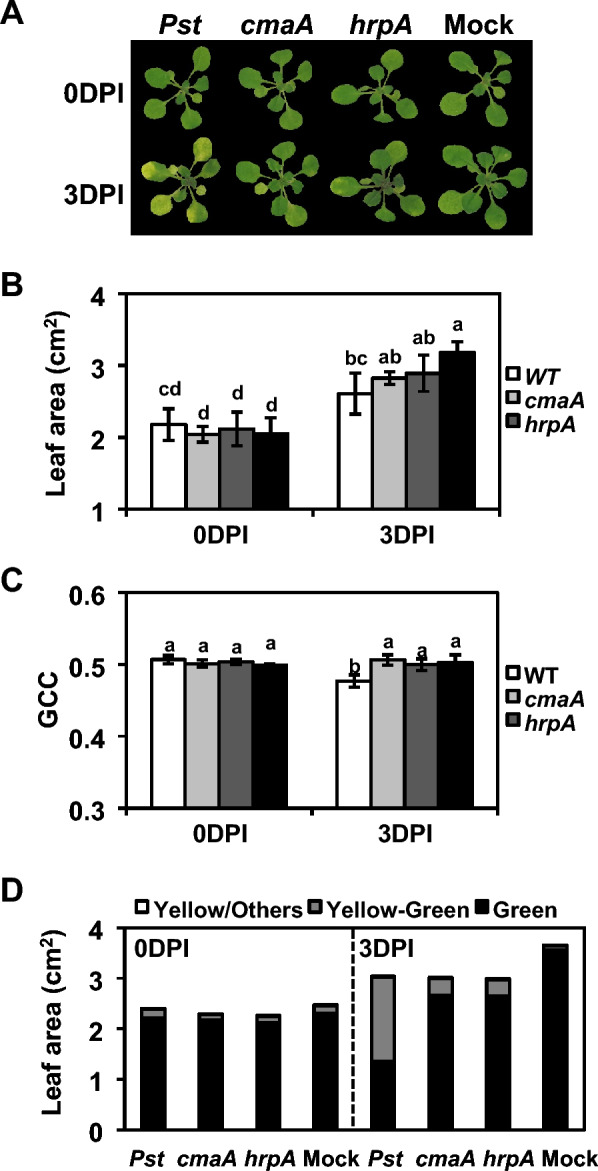
AraDQ-based image analysis of *Arabidopsis* seedlings treated with different *Pseudomonas* strains. Seedlings of *A. thaliana* (Col-0) were flood-inoculated with 1.0 × 10^6^ colony-forming units (CFUs)/ml of *Pseudomonas syringae* pv. *tomato* DC3000 wild-type (WT) and *cmaA* and *hrpA* mutant strains, and the disease symptoms were analyzed using AraDQ at 3 days post-inoculation (DPI). **A** Visual comparison of flood-inoculated seedlings after image correction. **B** Leaf area (cm^2^) of flood-inoculated seedlings. **C** Green chromatic coordinate (GCC) of flood-inoculated seedlings. **D** Hue saturation value (HSV)-based green/yellow color categorization of individual flood-inoculated seedlings. The values of leaf area and GCC represent the means calculated using data obtained from six different seedlings, and error bars indicate standard deviation. Statistical significance was determined by one-way analysis of variance (ANOVA) and Tukey’s honestly significant differences (HSD) tests. Mean values marked with the same letter do not differ significantly (*p* < 0.05). The data shown are representative of three independent experiments

The phytotoxin coronatine and the type III secretion system (T3SS) are the two major virulence factors of *Pst* DC3000. Coronatine contributes to bacterial invasion and disease symptom development, whereas T3SS is essential for overcoming plant defense responses [62, 63]. In this study, we tested two strains mutated for *cmaA* and *hrpA*, in which coronatine production and functional T3SS formation were completely abolished, respectively [63–65]. First, *Arabidopsis* Col-0 seedlings were flood-inoculated with 1 × 10^6^ colony-forming units (CFUs)/ml of the wild-type (WT) *Pst* DC3000 and the two mutant strains, and images of the seedlings were captured before and after inoculation. Visible chlorosis symptoms were observed only in the WT-treated seedlings at 3 days post-inoculation (DPI) (Fig. 4a). Although the *cmaA*- and *hrpA*-treated seedlings appeared morphologically similar to the negative control (mock-treated) seedlings, subtle changes in their size and color were detected by image analysis using AraDQ. In the mutant-treated seedlings at 3 DPI, clear decreases in leaf area were observed compared to that in the negative control; however, the differences were not statistically significant (Fig. 4b). Statistical issues can be easily resolved by increasing the sample size, as this analysis was conducted using only six seedlings. Treatment with the mutant strains appeared to have no effect on GCC (Fig. 4c), whereas green/yellow color categorization of individual seedlings based on the HSV color space showed a noticeable increase in yellow-green colored rosette area compared to that observed in the negative-control seedlings (Fig. 4d). On the other hand, the WT-treated seedlings at 3 DPI showed a significant reduction in leaf area and GCC, as well as a dramatic increase in yellow-green colored rosette area compared to that observed in the negative-control seedlings (Fig. 4b–d).

**Fig. 5:**
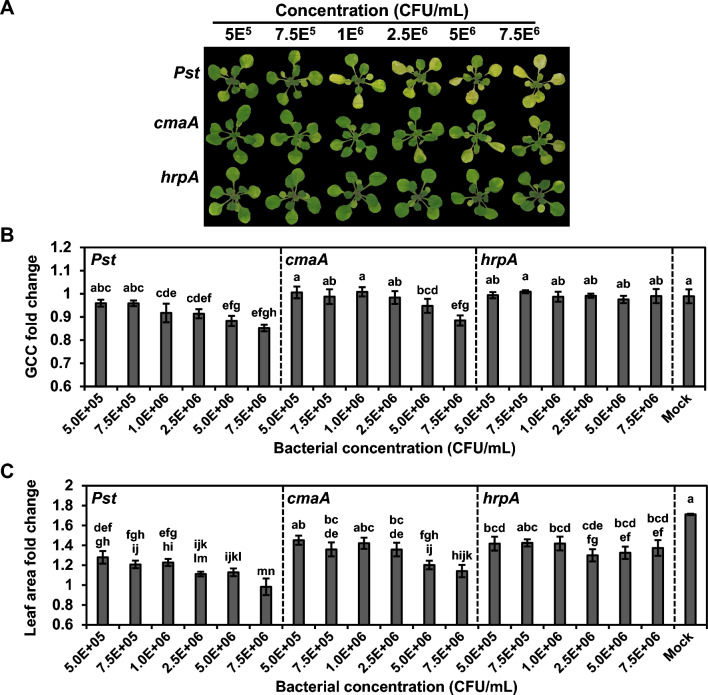
AraDQ-based image analysis of *Arabidopsis* seedlings treated with different concentrations of *Pseudomonas* strains. Seedlings of *A. thaliana* (Col-0) were flood-inoculated with six different concentrations of wild-type (WT) and *cmaA* and *hrpA* mutant strains of *P. syringae* pv. *tomato* DC3000, and the disease symptoms were analyzed using AraDQ at 3 days post-inoculation (DPI). **A** Visual comparison of flood-inoculated seedlings after normalization. **B** Fold changes in values of green chromatic coordinate (GCC). **C** Fold changes in values of leaf area (cm^2^). The fold change values represent the means calculated using data obtained from six different seedlings, and error bars indicate standard deviation. Statistical significance was determined by one-way analysis of variance (ANOVA) and Tukey’s honestly significant difference (HSD) tests. Mean values marked with the same letter do not differ significantly (*p* < 0.05). The data shown are representative of three independent experiments

To further determine plant size and color changes in response to the two mutant strains, we treated *Arabidopsis* Col-0 seedlings with varying concentrations of the bacterial suspension and analyzed their images using AraDQ (Fig. 5a). Fold changes in leaf area and GCC for each treatment were compared to minimize the effects of differences in the original states of the plants. As the inoculum concentration increased, a gradual decrease in projected leaf area and GCC fold change values was observed in the WT-treated seedlings, whereas the two mutant-treated seedlings exhibited different severities of disease symptoms (Fig. 5b, c). In terms of rosette greenness, treatment with the *hrpA* mutant did not cause a statistically significant reduction in GCC compared to that observed for the mock-treated seedlings at any concentration tested, whereas treatment with the *cmaA* mutant affected the GCC value at a concentration of 5.0 × 10^6^ CFU/ml (Fig. 5b). The fold change in GCC values of the *cmaA-*treated seedlings was similar to that of the WT-treated seedlings at a concentration of 7.5 × 10^6^ CFU/ml. In terms of rosette size, treatment with both *cmaA* and *hrpA* mutants at a concentration of 2.5 × 10^6^ CFU/ml reduced the seedling growth rate compared to that of the mock-treated seedlings (Fig. 5c). However, at inoculum concentrations higher than 2.5 × 10^6^ CFU/ml, *cmaA*-treated seedlings showed more severe growth retardation than that shown by *hrpA*-treated seedlings. Consistent with the results mentioned in previous reports [66, 67], these results indicated that T3SS is an essential virulence factor of *Pst* DC3000 and plays a more important role in pathogenesis than coronatine. These results also showed that image analysis using AraDQ is sensitive enough to detect non-visible alterations in the color and the morphology of diseased plants, as shown in a slight growth retardation of hrpA-treated seedlings without chlorosis symptoms likely due to the activation of plant basal defense response, and thus, AraDQ is capable of differentiating disease symptoms caused by various mutant strains. The image files used in this case study are provided in the released dataset on GitHub.

### Case study: analysis of disease symptoms in different lines of Arabidopsis

Engineering disease-resistant crops is a major strategy for sustainable food production [68]. *Arabidopsis* has served as a valuable model system to understand plant defense mechanisms, and these research efforts have been actively translated into development of disease-resistant crops [69–71]. Nevertheless, there are still many missing links in the understanding of defense signaling pathways [72]. To fill in these gaps in our knowledge of defense signaling pathways, genetic studies involving high-throughput quantitative evaluation of *Arabidopsis* lines with different levels of disease susceptibilities could be conducted.

**Fig. 6:**
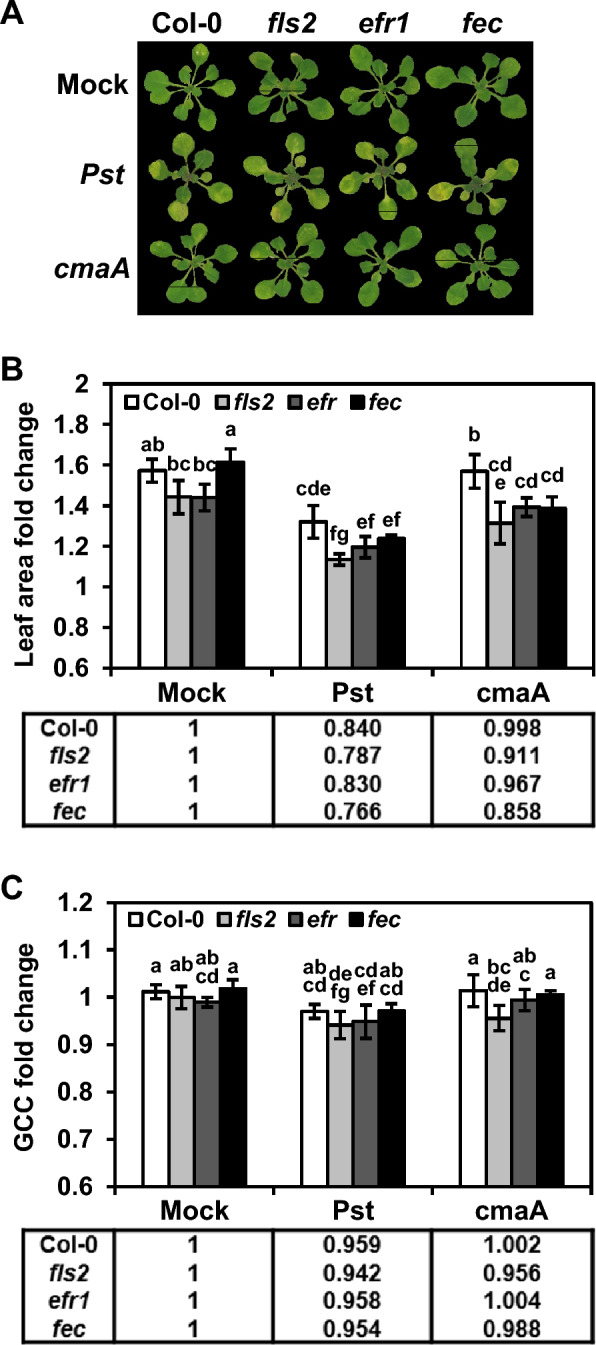
AraDQ-based image analysis of *Pseudomonas*-treated *Arabidopsis* seedlings having different genetic backgrounds. Seedlings of four different *A. thaliana* lines (Col-0, *fls2*, *efr*, and *fls2*/*efr*/*cerk1* [*fec*]) were flood-inoculated with suspensions containing 7.5 × 10^5^ colony-forming units (CFUs)/mL of wild-type (WT) and *cmaA* mutant strains of *P. syringae* pv. *tomato* DC3000, and their phenotypic responses were analyzed using AraDQ at 3 days post-inoculation (DPI). **A** Cropped images of flood-inoculated seedlings after normalization. **B** Fold changes in values of leaf area (cm^2^). The fold change values represent the means calculated using data obtained from six different seedlings. **C** Fold changes in values of green chromatic coordinate (GCC). The tables below each graph show that the relative fold change values for bacterial and mock treatments were estimated using the mean fold change values. Statistical significance was determined by one-way analysis of variance (ANOVA) and Tukey’s honestly significant difference (HSD) tests. Mean values marked with the same letter do not differ significantly (*p* < 0.05). Error bars indicate standard deviation. The data shown are representative of three independent experiments

In this study, we tested three *Arabidopsis* Col-0 mutant lines, *fls2*, *efr*, and *fls2*/*efr*/*cerk1*. These are mutants of pattern recognition receptors that sense conserved microbial molecules called pathogen-associated molecular patterns (PAMPs). FLS2, EFR, and CERK1 are localized to the plasma membrane and involved in the recognition of bacterial flagellin, bacterial elongation factor-Tu, and fungal chitin/bacterial peptidoglycan, respectively [73–75]. They play a central role in initiating PAMP-triggered immunity (PTI) as a basal defense response against a broad spectrum of microbial attacks [76]. To analyze changes in disease severity in the absence of full activation of PTI, the *Arabidopsis* Col-0 seedlings and the three mutant seedlings were flood-inoculated with suspensions (7.5 × 10^5^ CFU/ml concentration) of the WT and the *cmaA* mutant of *Pst* DC3000. At low inoculum concentrations, no clear differences between the Col-0 and the mutant lines were observed (Fig. 6a), although previous studies using bacterial growth assays have shown that enhanced disease susceptibility is observed in the *fls2* and the *efr* single mutants, and the highest level of susceptibility is observed in the *fls2*/*efr*/*cerk1* triple mutant [77, 78].

Interestingly, image analysis of the *Arabidopsis* mutant lines using AraDQ and comparison of the fold changes in values of rosette area and GCC for each seedling revealed alterations in the degree of disease susceptibility of the seedlings. To determine the effects of genetic variations, relative fold changes in values of rosette area and GCC of six bacteria- and mock-treated seedlings were calculated using the mean fold change in values observed for each *Arabidopsis* line for both parameters (Fig. 6b, c). In terms of the rosette area, the fold changes in values observed for the mock-treated seedlings were not statistically equivalent to each other, indicating that Col-0 and the mutant lines had different growth rates (Fig. 6b). The growth rate of the *fls2*/*efr*/*cerk1* triple mutant was higher than that of the *fls2* and *efr* single mutants but was similar to that of Col-0. All three mutant lines showed a significant reduction in fold changes in rosette area values compared to that of Col-0 under both WT- and *cmaA* mutant-treated conditions. Comparison of relative fold changes in values observed for pathogen-treated seedlings revealed that the *fls2*/*efr*/*cerk1* triple mutant showed the lowest growth rate, followed by that of *fls2*, *efr*, and Col-0. These findings are consistent with the results previously obtained from assays that were conducted to monitor bacterial populations, suggesting that the growth rate of seedlings under pathogen attack is closely associated with the level of disease susceptibility [77, 78]. In contrast, in terms of leaf greenness, all three treatments (mock-, WT-, and *cmaA* mutant-treatment) caused no significant differences in GCC values of Col-0 and the three mutant lines, although the WT-treated seedlings showed reduced GCC levels compared to those of the other seedlings (Fig. 6c). Relative fold changes in values of GCC also showed no clear pattern of reduction in Col-0 and the mutant lines, suggesting that leaf chlorosis severity was not significantly affected by defects in PTI defenses at low inoculum concentrations. Taken together, these results show that image analysis using AraDQ allows a quantitative comparison of disease severity, which in turn could help in validation and/or screening of alterations in the level of disease resistance in plants. The image files used in this case study are provided in the released dataset on GitHub.

## Conclusions

AraDQ is a user-friendly software for image-based phenotyping of flood-inoculated *Arabidopsis* seedlings. Its fully automated pipeline allows image preprocessing and data extraction from individual seedlings without the need for manual intervention. Image preparation for the use of AraDQ requires no or negligible setup costs, enabling easy adaptation to both routine and large-scale experiments. AraDQ measures various color-based and morphological parameters, which can be used for the phenotypic evaluation of plant disease severity under varying conditions of the disease triangle agents (host plant, pathogen, and environment). The potential applications of AraDQ include phenotyping of rosette plants (other than *Arabidopsis*) grown on an agar plate medium. The trained neural networks used in AraDQ can also be further adapted for analysis of plant images showing different growth stages or soil backgrounds.

## Availability and requirements

Project name: AraDQ-Arabidopsis Disease Quantification.

Project home page: https://github.com/kist-smartfarm/AraDQ.

Operating system(s): Windows, Mac OS, Linux.

Programming language: Python.

Other requirements: None.

License: Apache License 2.0, BSD 3-Clause “New” or “Revised” License.

Any restrictions to use by non-academics: None.

### Supplementary Information

**Additional file 1.** Primers used in this study. Primer sequences used for mutagenesis and confirmation of mutagenesis are listed in the table.

## Data Availability

The AraDQ software package, including the installation manual, and the datasets generated and analyzed during the current study are available in the GitHub repository at https://github.com/kist-smartfarm/AraDQ.

## Abbreviations

AraDQ: *Arabidopsis* disease quantification
CFU: Colony-forming unit
Col-0: *A. thaliana* Ecotype Columbia
CSV: Comma-separated values
DN: Digital number
DPI: Days post-inoculation
ExGI: Excess green index
FN: False-negative
FP: False-positive
GCC: Green chromatic coordinate
GRVI: Green and red ratio vegetation index
GUI: Graphical user interface
HSV: Hue saturation value
IoU: Intersection over Union
KB: King’s medium B
MES: 2-(N-morpholino)ethanesulfonic acid
MS: Murashige and Skoog
PAMP: Pathogen-associated molecular pattern
*Pst*: *P. syringae* Pv. *tomato*
PTI: PAMP-triggered immunity
qRT-PCR: Quantitative real-time polymerase chain reaction
RGB: Red, green, and blue color model
ROS: Reactive oxygen species
T3SS: Type III secretion system
TP: True-positive
VGG: Visual geometry group
VIA: VGG image annotator
WT: Wild-type
YOLO: You-Only-Look-Once
YOLOv4: YOLO version 4
