# Supplementary material for: AraDQ: an automated digital phenotyping software for quantifying disease symptoms of flood-inoculated Arabidopsis seedlings
Source: Plant Methods. 2024 Mar 16;20:44. doi: 10.1186/s13007-024-01171-w (PMC10943777; doi:10.1186/s13007-024-01171-w)
Supplement: Supplementary file 1 — Additional file 1. Primers used in this study. Primer sequences used for mutagenesis and confirmation of mutagenesis are listed in the table. [file 13007_2024_1171_MOESM1_ESM.docx]

**Additional file 1**. Primers used in this study.

| **Primers** | | **Sequences (5**′ **to 3**′**)** | |
| --- | --- | --- | --- |
| **Primers for mutagenesis** | | | |
| hrpA-F1 | GAGTCTTGATGAAAGGTTTG | |  |
| hrpA-F2 | GGTCGACGGATCCCCGGAATTTATTTCTGATTGCCCCCTC | |  |
| hrpA-R1 | GAAGCAGCTCCAGCCTACACAGATTTGATGCCCCTTAAGAT | |  |
| hrpA-R2 | GTCCTGATTGGACACGTTGC | |  |
| cmaA-F1 | CAATTCTCTTACCGCTATCC | |  |
| cmaA-F2 | GGTCGACGGATCCCCGGAATCTTTTCGTTGACGAAACAGG | |  |
| cmaA-R1 | GAAGCAGCTCCAGCCTACACATGAATGGCCCTCTGCTTGTG | |  |
| cmaA-R2 | GAACTCCGAGGTTTTCTCCG | |  |
| **Primers for confirmation of mutagenesis** | | | |
| Km1 | CAGTCATAGCCGAATAGCCT | |  |
| Km2 | CGGTGCCCTGAATGAACTGC | |  |
